# Tilorone mitigates the propagation of α-synucleinopathy in a midbrain-like organoid model

**DOI:** 10.1186/s12967-024-05551-7

**Published:** 2024-09-02

**Authors:** Qi Zhang, Meng Liu, Yue Xu, Juhyung Lee, Brothely Jones, Bing Li, Wenwei Huang, Yihong Ye, Wei Zheng

**Affiliations:** 1 Therapeutic Development Branch, National Center for Advancing Translational Sciences, National Institutes of Health, Bethesda, MD 20850 USA; 2 Cancer Data Science laboratory, National Cancer Institute, National Institutes of Health, Bethesda, MD 20892 USA; 3 Laboratory of Molecular Biology, National Institute of Diabetes, Digestive, and Kidney Diseases, National Institutes of Health, Bethesda, MD 20892 USA

**Keywords:** Tilorone, α-synuclein, Drug preclinical development, Midbrain-like organoid, Parkinson’s disease

## Abstract

**Background:**

Parkinson’s disease (PD) is a neurodegenerative condition characterized by the loss of dopaminergic neurons and the accumulation of Lewy-body protein aggregates containing misfolded α-synuclein (α-syn) in a phosphorylated form. The lack of effective models for drug screens has hindered drug development studies for PD. However, the recent development of in vitro brain-like organoids provides a new opportunity for evaluating therapeutic agents to slow the progression of this chronic disease.

**Methods:**

In this study, we used a 3D brain-like organoid model to investigate the potential of repurposing Tilorone, an anti-viral drug, for impeding the propagation of α-synucleinopathy. We assessed the effect of Tilorone on the uptake of fluorescently labeled α-syn preformed fibrils (sPFF) and sPFF-induced apoptosis using confocal microscopy. We also examined Tilorone’s impact on the phosphorylation of endogenous α-syn induced by pathogenic sPFF by immunoblotting midbrain-like organoid extracts. Additionally, quantitative RT-PCR and proteomic profiling of sPFF-treated organoids were conducted to evaluate the global impact of Tilorone treatment on tissue homeostasis in the 3D organoid model.

**Results:**

Tilorone inhibits the uptake of sPFF in both mouse primary neurons and human midbrain-like organoids. Tilorone also reduces the phosphorylation of endogenous α-syn induced by pathogenic α-syn fibrils and mitigates α-syn fibril-induced apoptosis in midbrain-like organoids. Proteomic profiling of fibril-treated organoids reveals substantial alterations in lipid homeostasis by α-syn fibrils, which are reversed by Tilorone treatment. Given its safety profile in clinics, Tilorone may be further developed as a therapeutic intervention to alleviate the propagation of synucleinopathy in PD patients.

**Supplementary Information:**

The online version contains supplementary material available at 10.1186/s12967-024-05551-7.

## Introduction

Parkinson’s disease (PD) is the second most prevalent neurodegenerative disorder after Alzheimer’s disease [1]. It predominantly affects the elderly population and is characterized by the degeneration of dopamine-producing cells in the substantia nigra, leading to decreased striatal dopamine levels, which in turn results in an imbalance of the direct and indirect basal ganglia pathways, causing motor symptoms such as bradykinesia, resting tremor, rigidity, and postural instability [2, 3]. Non-motor symptoms, including autonomic dysfunction, sleep disorders, and cognitive impairment, are also significant contributors to PD-associated disability. The etiology of PD is multifaceted, encompassing both genetic and environmental factors. The familial forms of the disease highlight the importance of genes such as *SNCA*, *LRRK2*, and *GBA*, while environmental factors include exposure to pesticides and a history of traumatic brain injury [4].

Current therapeutic strategies for PD primarily address symptomatic aspects. A primary pharmacological treatment is dopamine replacement therapy (DRT), which uses levodopa, a dopamine agonist, and monoamine oxidase-B (MAO-B) inhibitors. However, these treatments do not halt or slow the neurodegenerative process and are associated with side effects such as dyskinesia and motor fluctuations [5]. Thus, there is an urgent need for better PD therapies in clinical application.

A salient pathological hallmark of PD is the presence of intraneuronal proteinaceous inclusions known as Lewy bodies or Lewy neurites in the cell body and processes, respectively, in neurons. Alpha-synuclein (α-syn) is a key critical component of Lewy bodies and Lewy neurites [6]. In its native state, α-syn is a soluble, misfolding-prone protein predominantly found at presynaptic terminals, although its exact function remains elusive [7]. It has been implicated in physiological processes, such as synaptic vesicle trafficking and neurotransmitter release [8].

α-syn undergoes phosphorylation at serine 129, and the modified species is the predominant component in α-syn fibrils that form Lewy bodies in PD. Whether α-syn-containing aggregates is causal to neurodegeneration is unclear. However, α-syn aggregation is enhanced by PD-associated genetic mutations in *SNCA*, the gene encoding α-syn, and by post-translational modifications such as partial proteolysis [9]. Importantly, cell-to-cell propagation of α-syn-containing aggregates, a key feature of α-syn-associated PD pathology (also known as synucleinopathy), was observed in patient brains as the disease progressed. These findings led to the hypothesis that the prion-like transmission of α-syn aggregates, during which misfolded α-syn species acts as a template to seed and propagate neurotoxic conformers, might be a key contributing factor to this devastating disease [10–13].

In recent years, efforts have been intensified to develop disease-modifying treatments, with antibody-based and gene therapies being a significant focus. Antibodies can promote the clearance of amyloid-like aggregates or block their formation and propagation. Prasinezumab (PRX002) and BIIB054 are antibodies under investigation that target aggregated forms of α-syn. However, they face significant hurdles in clinical trials [14, 15]. Gene therapies that target α-syn directly, including strategies to knock down SNCA using antisense oligonucleotides (ASOs), are being tested with promising results observed in rodent and non-human primate models [16, 17]. However, these high-risk preclinical studies are time-consuming and resource-demanding. Economic in vitro platforms that allow rapid evaluation of drugs’ syncleinopathy-alleviating potential are urgently needed to accelerate PD drug development.

This study aims to develop a human induced pluripotent stem cell (iPSC)-derived 3D midbrain organoid system as an in vitro drug testing platform. We previously identified Tilorone as a potent inhibitor of heparan sulfate (HS)-mediated α-syn fibril uptake in 2D cell models [18]. Tilorone is a small molecule used in clinics as a broad-spectrum antiviral agent [19–22]. Studies have shown that Tilorone treatment stimulates interferon secretion in mice [23] and alters lysosome activities in vitro [24, 25]. Its newly established role in HS-mediated endocytosis prompted us to test Tilorone’s ability to inhibit α-syn fibril internalization and propagation in mature midbrain organoids, a 3D brain-like system. Our findings demonstrate that Tilorone inhibits effectively the cellular uptake of α-syn fibrils and suppresses fibril-induced endogenous α-syn phosphorylation in these midbrain-like organoids. Proteomic profiling of sPFF-treated organoids indicate that Tilorone can attenuate the abnormal cellular pathways associated with α-syn propagation. In conclusion, these results highlight the potential of Tilorone as a therapeutic agent for PD and underscore the advantages of using human 3D organoids in drug screening and preclinical development.

## Materials and methods

### Protein purification, labeling, and sPFF production

Human α-syn protein was expressed in BL21(DE3) RIL-competent *E. coli* and purified following an established protocol [26]. In brief, 1 L of *E. coli* culture was induced to express α-syn with 0.5 mM isopropyl β-d-1-thiogalactopyranoside at 20 °C overnight. Cells were pelleted by centrifugation at 5,000 rpm for 20 min, then resuspended in high-salt buffer (750 mM NaCl, 10 mM Tris pH 7.6, and 1 mM EDTA) with a protease inhibitor cocktail and 1 mM PMSF (50 mL for 1 L of culture). Resuspended cells were sonicated with a 0.25-inch probe tip at 60% power for a total time of 10 min. Sonicated cell lysate was boiled for 15 min to precipitate unwanted proteins and then cooled on ice for 20 min. The lysate was then centrifuged at 6,000 × g for 20 min. The supernatant containing α-syn was collected and dialyzed in TNE buffer (10 mM Tris pH 7.6, 25 mM NaCl, and 1 mM EDTA) overnight at 4 °C. Proteins were further fractionated using a Bio-Rad NGS chromatography system first through a size exclusion column in TNE buffer, followed by an ion exchange Mono-Q column. Protein was eluted in TNE buffer containing increasing concentrations of NaCl (25 mM to 1 M). Purified monomeric α-syn was dialyzed extensively in PBS, followed by labeling with a threefold molar excess of pH-Rodo Red or Alexa Fluor 596 succinimidyl ester fluorescence dye (Thermo Fisher) for 4 h at room temperature. Unconjugated dye was removed by dialysis in PBS. The labeled α-syn was adjusted to 5 mg/mL in a volume of 500 µL and then placed in a ThermoMixer shaker (Eppendorf) at 1,000 rpm at 37 °C for 7 d to generate α-syn preformed fibrils (sPFF). sPFFs were stained with 3% uranyl acetate and examined by electron microscopy to verify the formation of sPFF. PFFs were then stored in a − 80 °C freezer in small aliquots.

### Cells, immunostaining, immunoblotting, and data analyses

Wild-type C57BL/6 mice were obtained from Jackson Labs. The animal study protocol (K117-LMB-20) and ethical review were approved by the NIDDK Animal Research Advisory Committee. C57BL/6 mice were maintained under pathogen-free conditions in the Division of Veterinary Resources of National Institute of Health (NIH). Primary hippocampal neuron cultures were prepared from hippocampi of P0-1 murine pups. To obtain neuronal cells, hippocampi were dissected in Hanks’ balanced salt solution (HBSS) and washed with MEM (Thermo Fisher, 11095080) twice. The hippocampi were incubated with 0.25% trypsin-EDTA (Thermo Fisher, 25300062) containing 100 µg/mL DNase-I (Sigma, 10104159001) at 37 °C for 10 min. Trypsin was then inactivated by addition of MEM containing 10% FBS. After washing with MEM three times, the tissues were dissociated by pipetting several times in MEM containing 100 µg/mL DNase-I. Cells were then centrifuged at 300 x g for 5 min and resuspended in the plating medium (MEM containing 10% FBS, 1 mM sodium pyruvate (Sigma, S8636), 2 mM L-glutamine (Sigma, 59202 C), 100 µg/mL primocin (Invitrogen, ant-pm-05) and 0.6% glucose). The cell solution was passed through a 70-µm strainer (VWR, 76327–100) once to filter out any cell clumps. Cells were seeded into plates or µ-slide 4 well chamber with glass bottom (ibidi, 80,427) pre-coated with 50 µg/ml poly-D-lysine (Sigma, P7280) and 2 µg/mL laminin (Sigma, L2020). Generally, we seed hippocampal neuronal cells obtained from a pup to cover 4 cm^2^ or 8 cm^2^ of surface area for Immunoblotting and imaging purpose, respectively. We seed cortical cells obtained from a pup to cover 15 cm^2^ surface area for immunoblotting. After incubation at 37 °C with 5% CO_2_ for overnight, the culture medium was changed to the Neurobasal media (Thermo, 21103049) containing 2% B27 (Thermo, 17504044), 0.5 mM L-glutamine and 100 µg/mL primocin to support the growth of hippocampal neurons. The culture media was replaced with fresh media once a week or when needed.

Anti-mouse phosphorylated α-syn was stained with an antibody obtained from Abcam (catalog number: 51253). Generally, cells were fixed with 4% (wt/vol) paraformaldehyde/4% (wt/vol) sucrose/0.5% (vol/vol) TX-100 in PBS for 15 min, followed by primary antibody incubation overnight.

Anti-human phosphorylated α-syn antibody was obtained from Cell Signaling Technology (catalog number: 23706). Protein extraction was done from 5 to 4 pooled individual organoids using the 350 µl of 1× RIPA buffer (containing 50 mM Tris-HCl pH 7.4, 150 mM NaCl, 0.25% deoxycholic acid, 1% NP-40, 1 mM EDTA, protease inhibitor cocktail (Roche), and 1 x HaltTM phosphotase inhibitor (Thermo Fisher). The lysates were sonicated in the Q125 Sonicators (Thomas Scientific) for 10 cycles of 30 s on (20% output) and 30 s off. Then, samples were spin down at 12,000 g for 5 min at 4 °C. The 180 ul of supernatant were collected, mixed with 4 x loading buffer, and boiled at 95 °C for 5 min. Proteins in the samples were loaded into NuPAGE™ 4 to 12%, Bis-Tris gel (Thermo Fisher), followed by transferred to PVDF membrane. The membranes were blotted with antibody overnight and developed. Band quantifications were performed with Image Lab (Bio-Rad).

Fluorescence confocal images were acquired by a Nikon CSU-W1 SoRa microscope equipped with a temperature control enclosure and a CO_2_ control. 3D image reconstructions and analyses were done by Imaris software (Licensed to NIH). Fluorescence intensity was analyzed by open-source Fiji software. To this end, images were converted to individual channels, and regions of interest were drawn for measurement. Statistical analyses were performed using either Excel or GraphPad Prism 8.0 and 9.0. P values were calculated by Student’s t test using Excel or one-way ANOVA by GraphPad Prism 8.0 and 9.0. Linear curve fitting, nonlinear curve fitting, and IC50 calculation were done with GraphPad Prism 8.0 and 9.0. For nonlinear fitting, the inhibitor vs. response–variable slope model or the exponential decay model was used. Images were prepared by Photoshop and Illustrator (Adobe). Data processing and reporting are adherent to community standards.

### ATP content cytotoxicity assay in the 96-well format

cells were seeded in white, transparent bottom 96-well microplate (Thermo Fisher Scientific) at 20,000 cells per well in 100 µL/well neuronal medium and incubated at 37 °C with 5% CO2. The medium was carefully removed and 100 µL medium with compounds was added into each well. The plates were then incubated at 37 °C for additional 14 days. After incubation, 50 µL/well of ATPLite (PerkinElmer) was added to assay plates and incubated for 15 min at room temperature. The luminescence signal was measured using a Victor plate reader (PerkinElmer). Data were normalized with wells containing cells but no compound as 100%, and wells containing media-only as 0%.

### Midbrain organoid development

Midbrain organoids (MOs) were generated from iPSCs using a published method by Dexorgen, as government contracted service [27]. Briefly iPSCs were dissociated into single cells with Gentle Cell Dissociation Reagent (GCDR; Cat# 07174, STEMCELL, 100 ml/bottle) and plated into an Aggrewell plate (Stemcell Technologies Inc.) at 3 × 10^6^ cells per well. The plate was spun down according to manufacturer’s recommendation in containing midbrain differentiation medium supplemented with 5 µM ROCK inhibitor Y-27,632 (Tocris 1254). Spheroids were collected and transferred into an ultralow attachment 6-well plate (Corning) containing midbrain differentiation medium and placed on an orbital shaker rotating at 120 rpm in an incubator. After 18 days of differentiation, organoids were treated with 0.5 µM AraC for 14 days to stop the organoids from expanding too big. At day 60, organoids were transferred to a 12-well plated and treated with Tilorone (0, 0.5, 2, 4 µM; added 30 min prior to preformed fibrils) and 200 nM preformed fibrils. After 48 h of co-incubation, fresh medium with Tilorone alone was added to the organoids and cultured for an additional 14 days. Fresh medium was changed every other day and organoids were shaken at 120 rpm in an orbital shaker. On day 75, organoids were collected and fixed in 4% PFA and cryosectioned for immunostaining. Samples maintained, sliced and immunostained by Dexorgen, Inc as government contracted service.

### qPCR and proteomic analyses

Taq man Parkinson’s disease associated genes array plates were purchased from Thermo Fisher with array ID: PRPRJ2W. Total RNA was extracted from 10 brain organoid per each sample using TriPure reagent (Roche) and purified using RNeasy MinElute Cleanup Kit (Promega) following the standard protocols. The RNA concentration was measured by Nanodrop 2000 UV spectrophotometer, and 1 µg total RNA was converted to cDNA using the iScript Reverse Transcription Supermix (BioRad) system. Samples were analyzed on ViiA 7 real-time PCR system with standard protocol (Table S1). For proteomic sequencing, the service was provided by Poochon scientific as government contract service. Generally, 18 frozen organoids pellet samples were received and processed for the study. The preparation of the protein lysates from 18 frozen pellet samples was performed. Protein concentrations were measured using the BCA method. An equal amount of total protein from each sample was processed for MS analysis. In summary, 100 µg of protein from each of the 18 samples was processed for trypsin digestion, followed by TMT-multiplex labeling using one TMT-16plex set. 18 unique TMT tags were used to label 50 µg of trypsin-digested peptides from each of the 18 digests. The LC-MS/MS analysis was carried out using a Thermo Scientific Orbitrap Exploris 240 Mass Spectrometer and a Thermo Dionex UltiMate 3000 RSLCnano System. Each peptide fraction from the set of 24 fractions was loaded onto a peptide trap cartridge at a flow rate of 5 µL/min. The trapped peptides were eluted onto a reversed-phase 25 cm C18 EasySpray nano column (Thermo) using a linear gradient of acetonitrile (3–36%) in 0.1% formic acid. The elution duration was 110 min at a flow rate of 0.3 µL/min. Eluted peptides from the EasySpray column were ionized and sprayed into the mass spectrometer, using an EasySpray Ion Source (Thermo) under the following settings: spray voltage, 1.6 kV, Capillary temperature, 275 °C. The 24 fractions were analyzed sequentially.

The Exploris 240 instrument was operated in the data dependent mode to automatically switch between full scan MS and MS/MS acquisition. Survey full scan MS spectra (m/z 350 − 1800) was acquired in the Orbitrap with 35,000 resolutions (m/z 200) after an accumulation of ions to a 3 × 10^6^ target value based on predictive automatic gain control (AGC). The maximum injection time was set to 100 ms. The 20 most intense charged ions (z ≥ 2) were sequentially isolated and fragmented in the octopole collision cell by higher-energy collisional dissociation (HCD) using normalized HCD collision energy at 30% with an AGC target of 1 × 105 and a maximum injection time of 400 ms at 17,500 resolutions. The isolation window was set to 2 and fixed first mass was 100 m/z. The dynamic exclusion was set to 20 s. Charge state screening was enabled to reject unassigned and 1+, 7+, 8+, and > 8 + ions.The set of 24 MS Raw data files acquired from analysis of the 24 fractions were searched against human protein sequences database obtained from the Uniprot KB website using Proteome Discoverer 2.4 software (Thermo, San Jose, CA) based on the SEQUEST and percolator algorithms. The false positive discovery rate (FDR) was set to 1%. The resulting Proteome Discoverer Report contains all assembled proteins with peptides sequences and peptide spectrum match counts (PSM#), and TMT-tag based quantification ratio.

### Proteomics data analysis

Foldchanges of protein abundance were calculated as ratio of mean protein expression under compared conditions (Table S2). P-values were calculated by Student T test. Proteins with log2Foldchanges > 0.1 and p-values < 0.05 were considered as differentially expressed proteins. R package ggplot2 (version 3.4.0) was used to plot the volcano plot of differentially expressed proteins. R package pheatmap (version 1.0.12) was used to plot all the heatmap figures.

### Gene ontology (GO) analysis

Genes with average fold changes ≥ 1.5 were selected for Gene Ontology (GO) analysis of biological pathways (BP) and cellular components (CC) using function enrichGO from R package ClusterProfilter (version 4.2.2). Terms with adjusted p-values < 0.05 were considered as significantly enriched biological terms. Top 30 significantly enriched biological terms ordered by adjusted p-values were displayed. R package aPEAR (version 1.0.0) was used to calculate enrichment networks of the top 30 significantly enriched terms. The GO pathway analyses were obtained from GeneCards (https://www.genecards.org/).

## Results

### Tilorone inhibits α-syn fibril uptake and fibril-induced endogenous α-syn phosphorylation in primary neurons

Our recent drug repurposing screen identified the antiviral drug Tilorone as a small-molecule inhibitor that blocks cellular uptake of heparan sulfate (HS)-mediated cargos including α-syn sPFF [18]. The screening, however, was performed using an immortalized cancer cell line. Thus, it is unclear whether Tilorone can inhibit the cell entry of α-syn sPFF in neuronal cells.

**Fig. 1:**
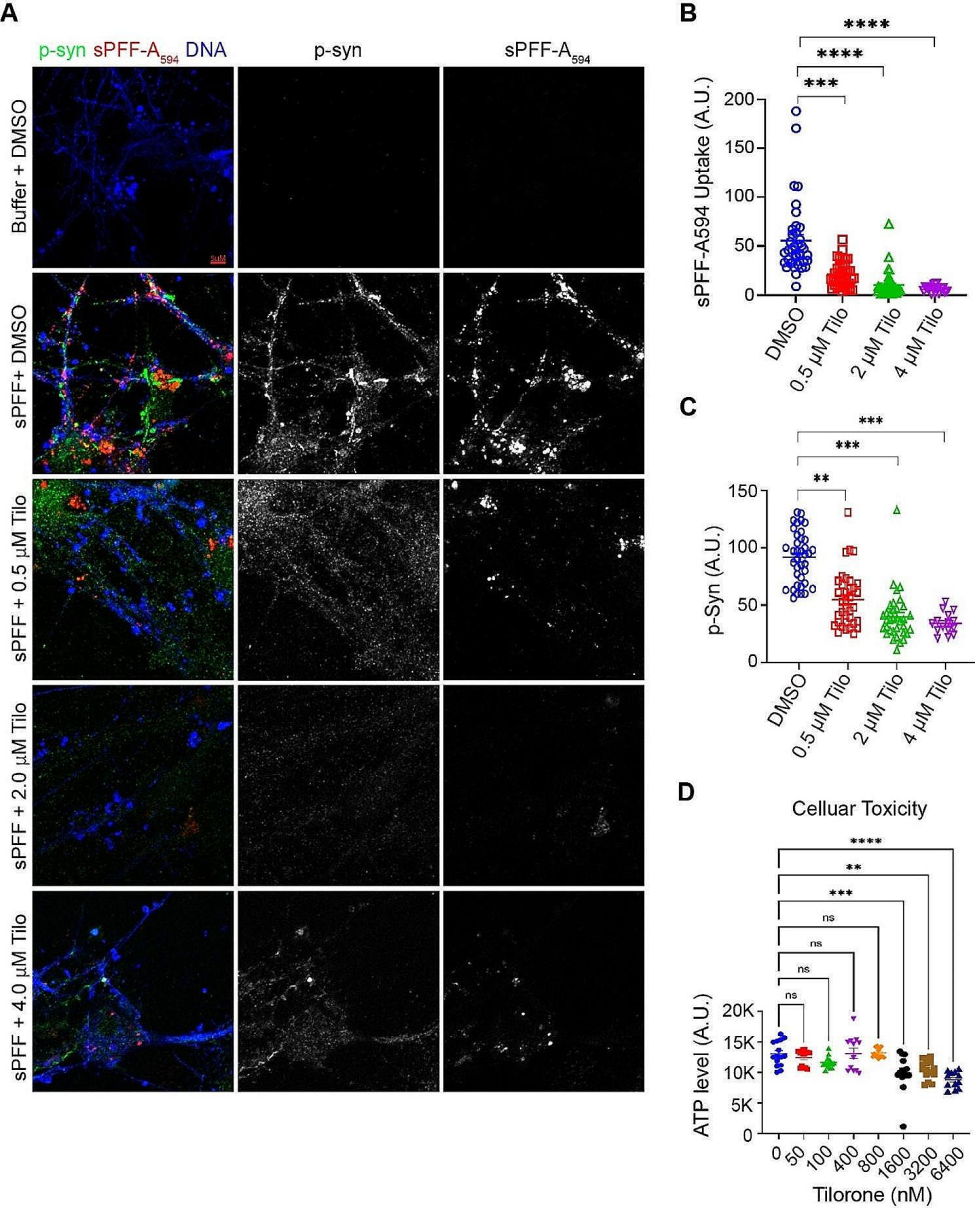
Tilorone reduces α-syn fibril-induced phosphorylation of endogenous α-syn in primary neurons. **(A)** Primary neurons treated with Alexa Flour _594_-labeled preformed human α-syn fibrils (sPFF-A_594_) (100 nM) for 14 days in the absence or presence of the indicated concentrations of Tilorone (Tilo.) were stained with antibodies specifically against phosphorylated mouse α-syn in green. **(B)** Quantification of internalized sPFF-A_594_. A.U., arbitrary units. **(C)** Quantification of phosphorylated α-syn fluorescence intensity. **(D)** Primary neurons treated with varied doses of Tilorone for 14 days, and the cellular ATP level was measured by an ATPLite luminescence assay. (*n* = 3, Ordinary one way ANOVA analysis, *p* value not significant for 50 nM sample vs. untreated, 100 nM vs. untreated, 400 nM vs. untreated, 800 nM vs. untreated, *p* value 0.0001 for 1600 nM vs. untreated, *p* value 0.0018 for 3200 nM vs. untreated, *p* value < 0.001 for 6400 nM vs. untreated)

To evaluate the effect of Tilorone on neurons, we isolated primary hippocampal neurons from newborn mice and exposed them to fluorescence-labeled α-syn sPFF in the absence or presence of Tilorone at different concentrations for two weeks. Confocal fluorescence microscopy confirmed that Tilorone effectively decreased the uptake of α-syn fibrils by mouse primary neurons in a dose-dependent manner, with maximal inhibition observed at two micromolars (Fig. 1A, B). Moreover, by immunostaining cells with an antibody recognizing Ser129-phosphorylated α-syn, which is known to be induced by exogenous α-syn fibrils, and is a major Lewy body component and a disease progression marker, we demonstrated that Tilorone could also reduce sPFF-induced endogenous α-syn phosphorylation (Fig. 1A, C).

We also assessed the cytotoxicity of Tilorone on these neurons by titrating its concentration from 50 nM to 6.4 µM using an assay monitoring cellular ATP levels (Fig. 1D). Tilorone did not affect the cellular ATP levels at concentrations below 1 µM. Collectively, these findings indicate that Tilorone can mitigate α-syn fibril uptake and seeding by neurons at concentrations well tolerated by neurons.

### An organoid culture system recapitulates PD pathology

The human midbrain-like organoids offers advanced three-dimensional (3D) models that mimic the complex tissue structures formed by different cell types in the human midbrain. Derived from human iPSC, these organoids provide a powerful platform for detailed investigation of mechanisms underlying neurological disorders such as PD.

**Fig. 2:**
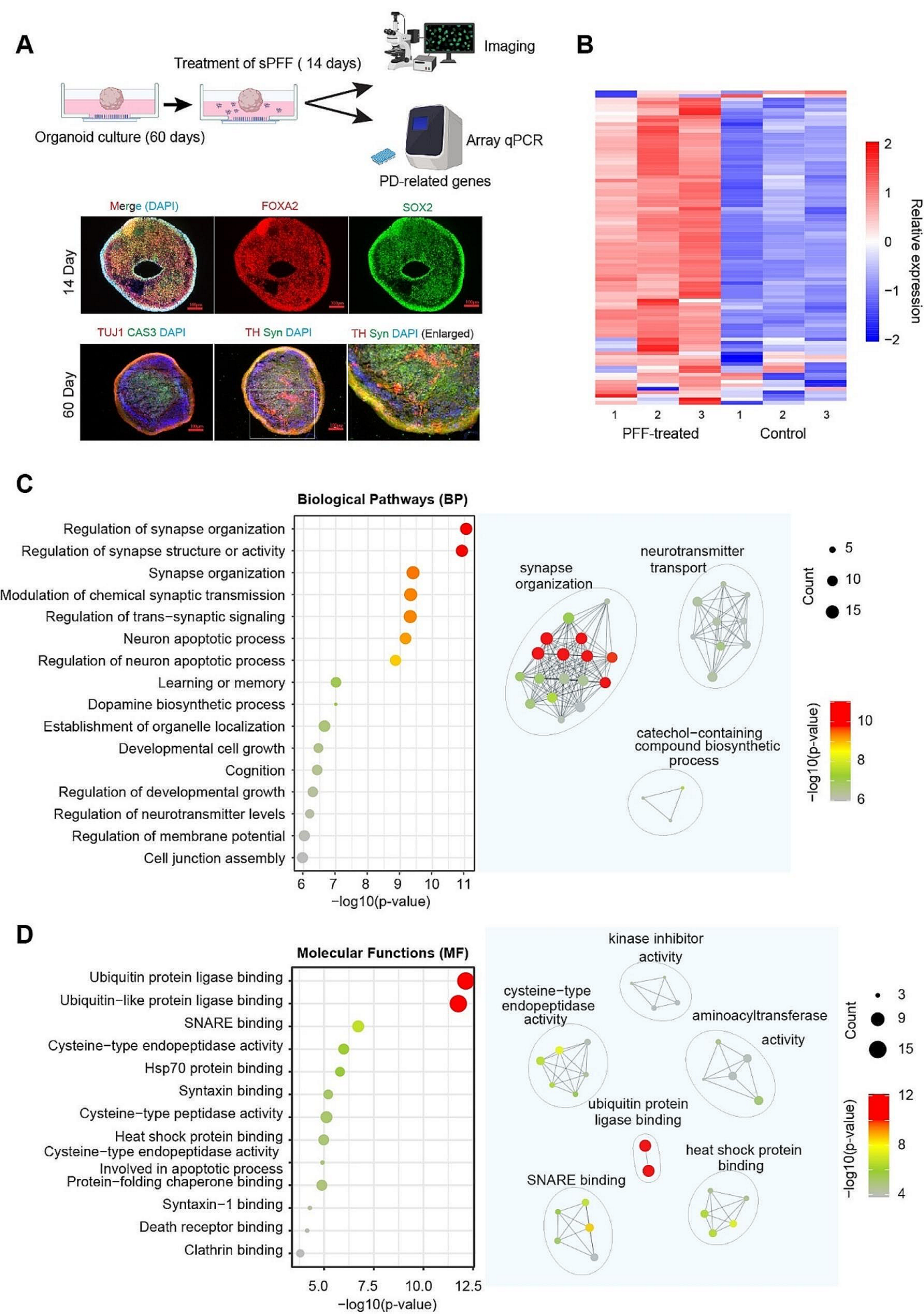
Upregulated PD-replated genes in 3D organoids treated with preformed α-syn fibrils. **(A)** The experiment scheme for assessing the midbrain-like organoids treated with preformed α-syn fibrils and Tilorone (top). The bottom panels show representative images of differentiated organoids immunostained by antibodies against the indicated neuronal markers at different time points. Note that midbrain-like organoids became mature at day 60. **(B)** A heatmap shows the relative expression levels of 94 PD**-**associated genes before and after sPFF treatment. **(C)** Enriched Biological Pathways (BP) or **(D)** molecular functions (MF) of the upregulated genes (*n* = 75, fold change ≥ 1.5-fold) analyzed by RT-qPCR. GO terms with p-values < 0.05 were considered as significantly enriched terms. Highly enriched terms were displayed in the dot plot (left) with each dot representing an enriched GO term. The size of the dot indicates gene number. The dot color indicates the level of significance. The top 30 highly enriched GO terms were used to identify enriched networks (right). GO terms were clustered based on the similarity of the enriched genes in the terms. GO terms were clustered and connected by lines if they share enriched genes. Shorter lines between GO terms (represented by dots) indicate higher similarity of enriched genes in these terms

To develop a midbrain-like PD-relevant organoid model for drug testing, we cultured and differentiated iPSC in 3D. We cultured these organoids for 60 days to achieve sufficient maturation. Immunostaining and confocal microscopy confirmed the developments of dopaminergic neurons and other neuronal cell types at this time point (Fig. 2A).

We then subjected the organoids to a treatment regime involving sPFF for additional 14 days. We analyzed the expression levels of 94 PD-associated genes by microarray-based quantitative RT-PCR (Fig. 2A). Significantly altered genes were analyzed to identify interactions based on either biological pathways or molecular functions. These analyses revealed a significant upregulation of several modules such as synaptic formation, synapse structure or activity regulation, and ubiquitin protein ligase binding as molecular signatures altered by α-syn fibrils. These changes might result from an adaptive response to the proteotoxic insult by cells in the organoids to maintain neuronal communications and functions, which may not be entirely successful because our gene expression analysis also suggested a modest elevation in neuronal apoptotic processes following α-syn fibril treatment (Fig. 2B, C). Thus, the results of our array-based qRT-PCR suggest that organoids exposed to α-syn fibrils do exhibit some features of PD pathology, confirming them as a valuable tool for PD therapeutic evaluation.

### Tilorone inhibits α-syn fibrils uptake and fibril-induced α-syn hyper-phosphorylation in human midbrain-like organoids

**Fig. 3:**
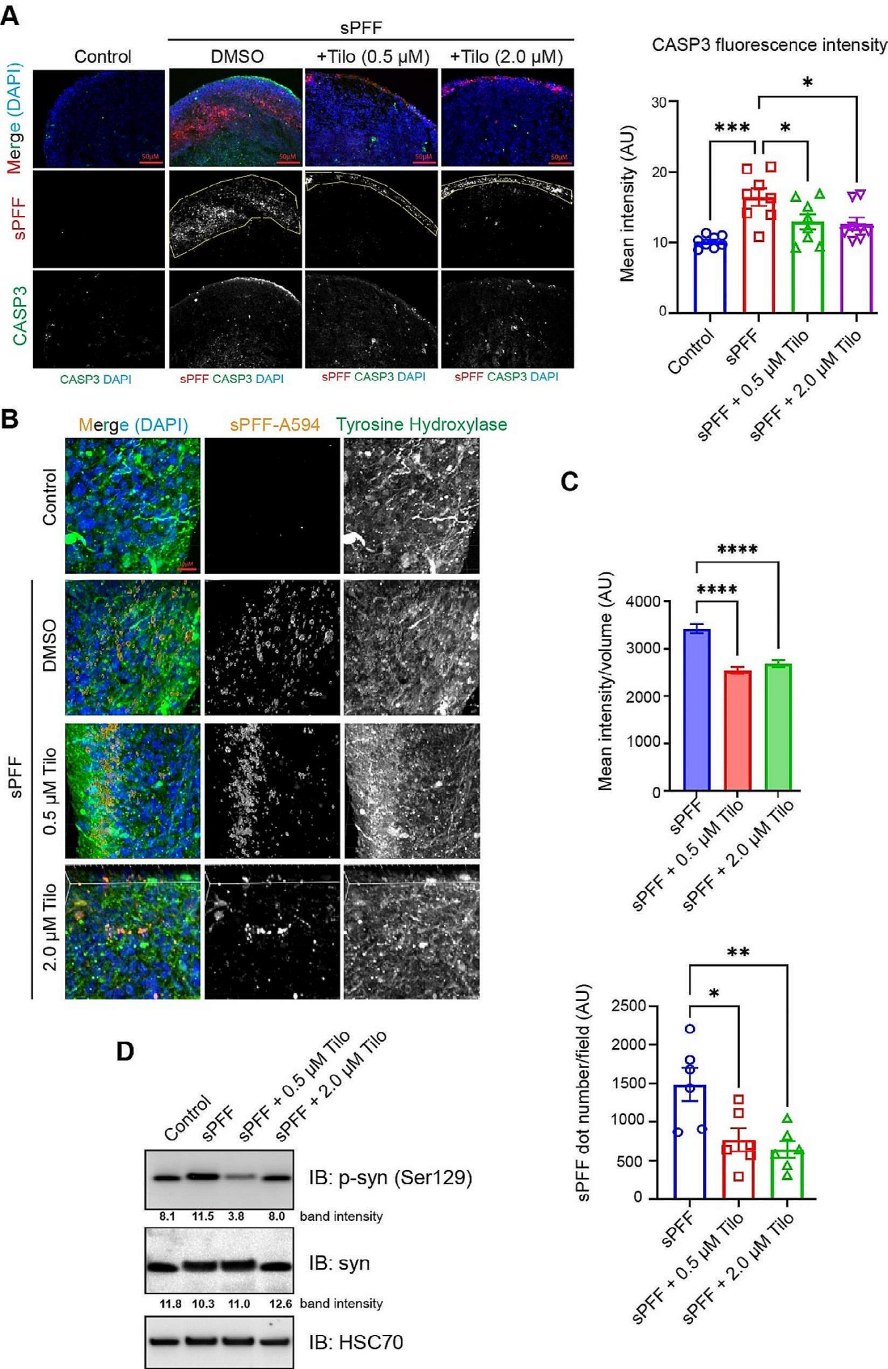
Tilorone reduces sPFF-induced pathology in midbrain-like organoids. **(A)** Tilorone reduces cell death induced by added α-syn fibrils. **(B)** Organoids treated with Alexa Fluor_594_-labeled sPFF (orange) and Tilorone were sliced and stained with antibody targeting tyrosine hydroxylase (Green). Images were taken by 3D super-resolution fluorescence microscopy. **(C)** The graphs show the quantification of internalized sPFF fluorescence intensity averaged by spot volume (top) and the quantification of internalized sPFF spot number averaged by field (*n* = 2 Ordinary one-way ANOVA analysis, *p* value < 0.001 for 0.5µM vs. Ctrl, *p* value < 0.001 for 1µM vs. Ctrl). **(D)** Tilorone reduces sPFF-induced hyper-phosphorylation of α-syn in 3D organoids as revealed by immunoblotting

To investigate Tilorone’s impact on synucleinopathy in midbrain-like organoids, we administered co-treatments involving fluorescence-labeled sPFF and different concentrations of Tilorone. Consistent with our gene expression analyses (Fig. 3), immunostaining and confocal microscopy revealed that α-syn fibril treatment accelerated cell death in the organoid’s outermost layers, as shown by the cell death marker Caspase3 (Fig. 3A). Notably, the distribution of α-syn fibrils within Tilorone-treated organoids diverged significantly from those treated with DMSO control. While control organoids exhibited diffusive α-syn fibril signals in a thick layer of cells, Tilorone treatment resulted in the concentration of fibrils primarily in a thin layer at the organoid’s periphery (Fig. 3A, B). This suggests Tilorone not only impedes the internalization of fibrils but also restricts their intercellular transmission within the organoid. As expected, 3D confocal microscopy analyses of sliced organoid samples confirmed reduced uptake of α-syn fibrils by the organoids, upon Tilorone treatment (Fig. 3C).

We next employed antibodies specific to human α-syn phosphorylated at Ser129 to analyze whole-cell extract samples from organoids exposed to α-syn fibrils in the presence or absence of Tilorone. The results indicated a modest increase in α-syn phosphorylation in organoids treated solely with sPFF compared to the untreated control group. Importantly, Tilorone co-treatment led to a marked reduction of phosphor-α-syn, notably at a concentration of 0.5µM (Fig. 3D).

### Proteomic analyses reveal disease-associated signatures rescued by Tilorone

**Fig. 4:**
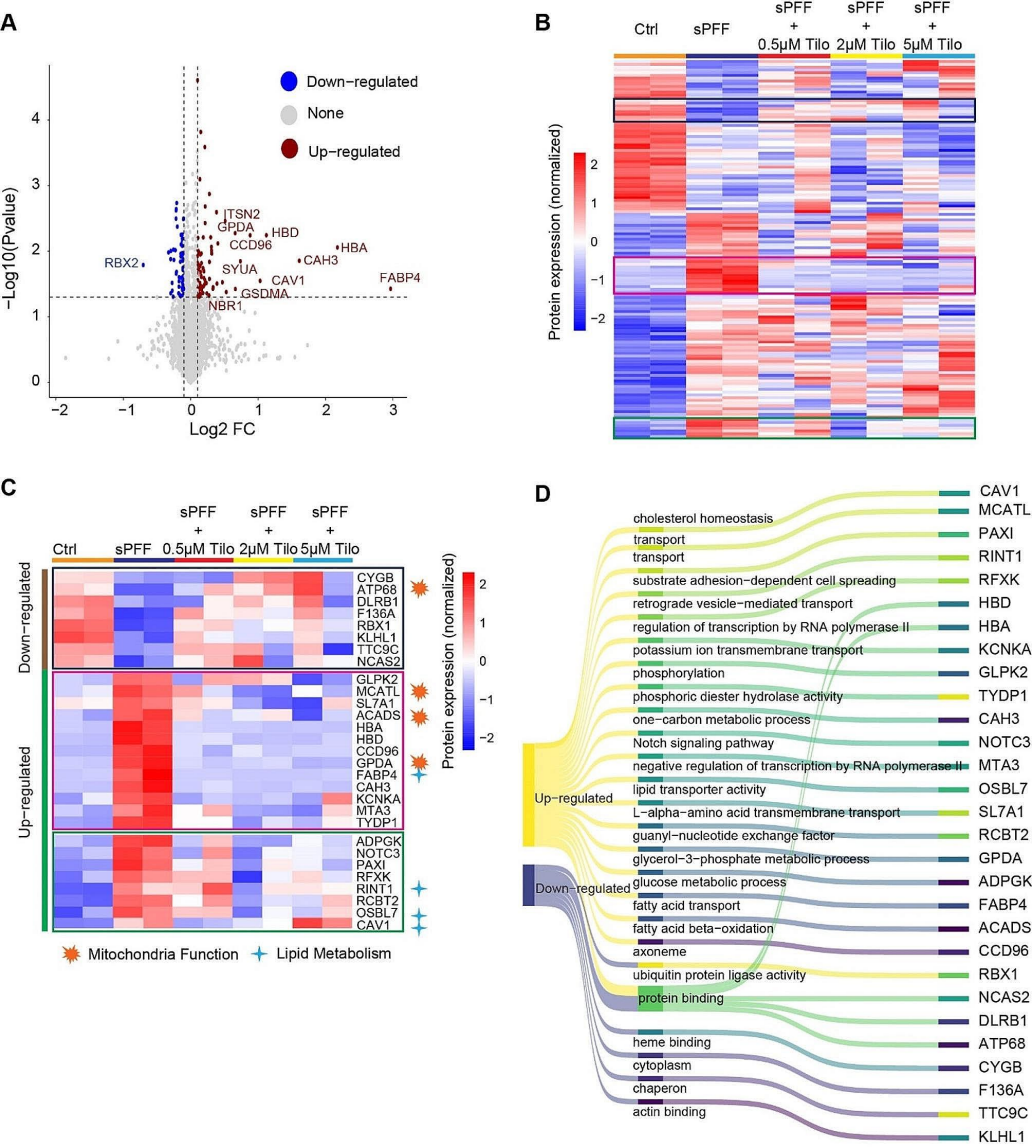
The effect of Tilorone on the global proteome of sPFF-treated organoids. **(A)** A volcano plot shows the proteins differentially regulated in sPFF-treated organoids. Dashed lines denote the threshold of p-value < 0.05 and Log2 FoldChange > 0.1. Proteins with p-values < 0.05 and Log2 FoldChange > = or < = 0.1 were color-labeled, in red and blue, respectively. **(B**,** C)** Heatmaps showing proteins whose abundance was altered by sPFF and Tilorone (Tilo). Proteins with p-value < 0.05 and Log2 FoldChange > 0.1 were considered as differentially regulated. B shows the relative abundance of all differentially regulated proteins. C highlights proteins indicated in the boxed area in B. **(D)** A Sankey plot showing involved biological pathways of the proteins in C. The Gene Ontology (GO) results for each protein are obtained from GeneCard.

To have an in-depth understanding of how Tilorone affects the response of organoids to α-syn fibrils, we extracted proteins from untreated, α-syn fibril-treated organoids and those co-treated with α-syn fibrils and Tilorone. We used mass spectrometry to analyze these samples’ whole proteome and compared the relative abundance of the identified proteins. Unlike array-based qRT-PCR analyses (Fig. 2), this approach is unbiased and more thorough. Indeed, our analyses revealed a profound change in the whole proteome between untreated and α-syn fibril-treated organoids (Fig. 4A). Ontology pathway analysis suggested that many proteins related to lipid metabolism (e.g., FABP4, OSBPL7, RINT1, and CAV-1) were upregulated by sPFF (Fig. 4B-D), strengthening a previously suggested link between lipid deregulation and PD pathology [28, 29]. Additionally, several proteins critical for mitochondrial functions are significantly altered by α-syn fibril treatment (Fig. 4B-D), recapturing the role of mitochondria in PD pathology.

Among proteins affected by α-syn fibril treatment, only a small subset was reversed by exposing α-syn fibril-treated organoids to Tilorone. Notably, the most significant list includes many of the above-mentioned proteins involved in lipid metabolism and mitochondria regulation (Fig. 4B, C). For example, fatty acid binding protein 4 (FABP4), a fatty acid transporting protein that regulates lipid homeostasis [30, 31], was most significantly upregulated by α-syn fibril treatment, and this upregulation was completely abolished by Tilorone co-treatment (Fig. 4C). Other fibril-induced proteins whose abundance was reduced by Tilorone include OSBPL7, which belongs to the oxysterol-binding protein-like family known for their roles in intracellular lipid transfer at membrane contact sites [32–34], Caveolin-1 (CAV-1), a caveolae-associated membrane protein widely associated with endocytosis, extracellular matrix organization, and cholesterol distribution [35], and Rad50 interacting protein (RINT1), which regulates vesicular trafficking between the ER and Golgi apparatus and lipid droplet biogenesis [36]. Among the identified mitochondria regulators, Acyl-CoA Dehydrogenase Short Chain (ACADS) is crucial for converting short-chain fatty acids into acetyl-CoA, feeding into the citric acid cycle for ATP production at the mitochondria. Glycerol-3-Phosphate Dehydrogenase 1 (GPDA), involved in the glycerol-phosphate shuttling, facilitates the transfer of reducing equivalents (NADH) from the cytosol into the mitochondria, supporting ATP production through oxidative phosphorylation. Mitochondrial basic amino acids transporter (MCATL) carries amino acids, carboxylic acids, fatty acids, and nucleotides across the inner membrane and is crucial for energy conversion [37, 38].

### Tilorone suppresses neuroinflammation in sPFF-treated organoids

**Fig. 5:**
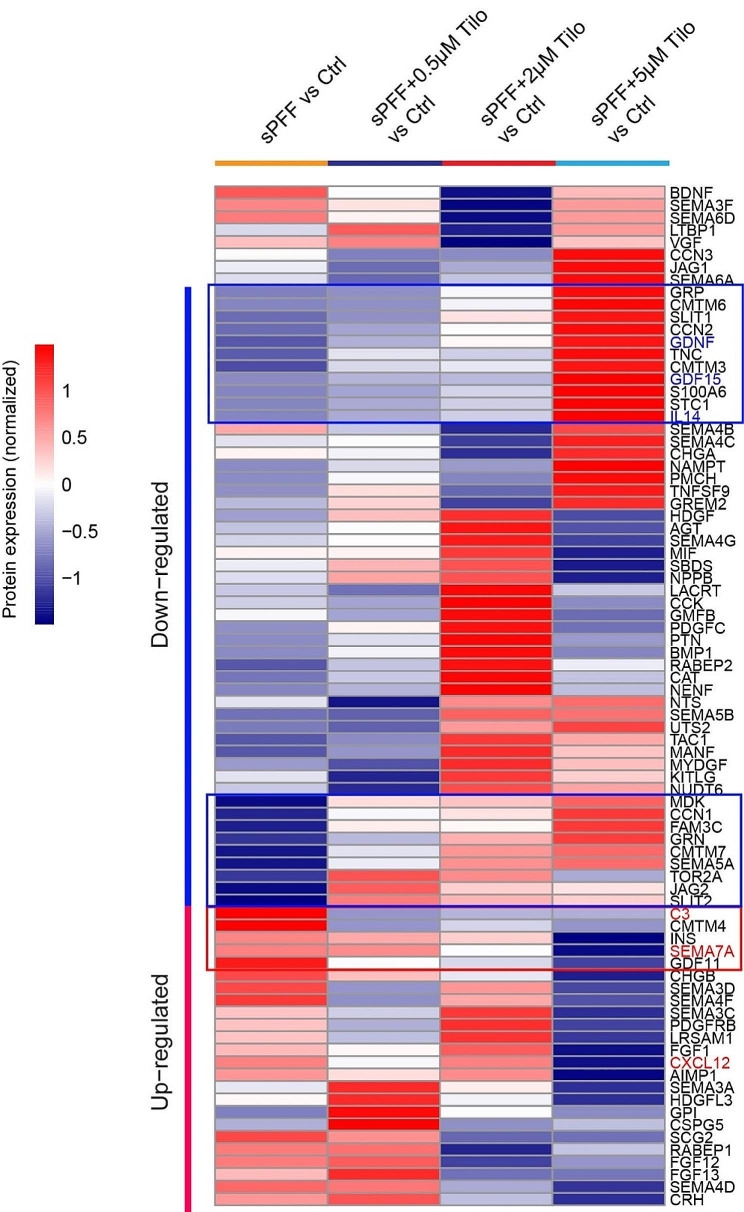
The effect of Tilorone on secretory proteins in sPFF-treated midbrain organoids. A heatmap showing secretory proteins whose abundance was altered by sPFF and Tilorone (Tilo). Proteins with p-value < 0.05 and Log2 FoldChange > 0.1 were considered as differentially regulated

Since previous studies revealed signs of neuroinflammation in sPFF-treated neurons [39, 40]and because Tilorone was known to activate interferon production in mice [41], we examined whether Tilorone could affect neuroinflammation in sPFF-treated organoids. To this end, we re-analyzed the mass spectrometry data, focusing on the expression of a collection of secretory proteins including cytokines and chemokines in midbrain organoids treated with sPFF or with both sPFF and Tilorone. When we compared the protein levels of these treated organoids to these of untreated control organoids, only a few neuroinflammation-related proteins were detected, likely due to efficient secretion. Nevertheless, our analysis showed that these proteins, including complement component 3 (C3), SEMA7A, and CXCL12, were among the most significantly upregulated ones following sPFF treatment (Fig. 5), suggesting that sPFF indeed induces neuroinflammation in organoids. Strikingly, in organoids co-treated with Tilorone and sPFF, these proteins were either restored to near-normal levels or further downregulated. Mass spectrometry data also showed that sPFF treatment downregulated a cohort of secretory proteins closely linked to neuronal functions. These include Interleukin-14 (IL-14, also known as α-taxilin), a syntaxin-binding protein known to regulate vesicle fusion and neurotransmitter release [42], GDF15, a neuroprotective factor [43], and GDNF (Glial Cell-Derived Neurotrophic Factor), a critical regulator of dopaminergic neuron growth and survival [44, 45]. To our satisfaction, Tilorone treatment dose-dependently elevated these proteins, highlighting its role in counteracting the neurodegenerative activities of α-syn fibrils (Fig. 5). These findings underscore the potential of Tilorone to regulate key cytokines involved in neuronal health, offering new insights into the therapeutic strategies for PD. Our analyses did not identify interferons even in cells treated with a high dose of Tilorone either by itself or with sPFF (Fig. 5, Figure S1), which might be due to low expression of these factors under non-infection conditions or efficient secretion.

## Discussion

Our study suggests a potential neuroprotective role for Tilorone in mitigating synucleinopathy-associated with PD because it effectively reduces the uptake of α-syn fibrils and the propagation of phosphorylated α-syn in human midbrain-like organoids. This is particularly significant given that phospho-α-syn is a major component of Lewy bodies implicated in a toxic gain-of-function leading to neuronal cell death. The reduction of fibril uptake and α-syn phosphorylation by Tilorone supports it as a therapeutic agent targeting early stages of synucleinopathy-associated neuronal damages.

Tilorone is currently used in clinics as an anti-viral agent in several European countries [19–22]. It can be taken orally and has a broad anti-viral spectrum [46]. It was suggested to act as an immune modulator, inducing the production of anti-viral cytokines such as interferon [23]. Additional studies suggest that it may also serve as a lysosomotropic agent, altering the pH of endolyososomes to influence viral entry [24, 25]. This mode of action is consistent with Tilorone being an α-syn endocytosis inhibitor, underscoring a common mechanism exploiting the vulnerability of a host endocytic system for delivering misfolded protein aggregates and viruses [47].

The utilization of brain-like 3D organoids to model key aspects of PD pathology provides a new tool in PD therapeutic development. When mature organoids were exposed to α-syn fibrils, we observed a notable upregulation in synaptic maintenance function and ubiquitin ligase-binding activities (Fig. 2C). These changes suggest an enhanced homeostasis activity in these tissues, potentially as an adaptive response to the proteotoxic stress. Ubiquitin ligases are pivotal in targeting misfolded or damaged proteins for degradation via the proteasome or lysosomes. The increased ubiquitin-binding activity may indicate a cellular attempt to control the accumulation of α-syn fibrils, which are detrimental to neurons. Thus, this upregulation might reflect an intrinsic neuroprotective mechanism to mitigate the potentially harmful effects of fibril accumulation.

Our proteomic analyses also illuminate how 3D brain organoids respond to pathogenic α-syn fibrils and how Tilorone modulates these responses. The most deregulated biological signatures by α-syn fibrils are linked to lipid metabolism and mitochondria functions, which have been implicated in PD pathogenesis [48]. Specifically, the fatty acid binding protein FABP4 upregulation is associated with microglia-associated inflammation and apoptosis in various cell types [49–51]. FABP4 was also suggested as one of the plasma biomarkers in early-onset PD patients and PD [52, 53]. The modulation of FABP4 by sPFF may disrupt lipid homeostasis to potentiate neuroinflammation. Indeed, our study has revealed the upregulation of several neuroinflammation-related proteins by sPFF, which may depend on FABP4 induction since inhibition of FABP4 was shown to reduce inflammation and apoptosis in models of kidney injury and in cells exposed to lipopolysaccharide [54, 55]. Our findings suggest the possibility of using Tilorone to suppress neuroinflammation and restore the decline of the proteostasis network comprised of proteins such as FABP4, OSBPL7, RINT1, and CAV-1, which are integral to lipid homeostasis and cell viability. However, the beneficial effect of Tilorone may depend on its concentration because in organoids treated with Tilorone, a two-fold difference in dosing can result in a drastically different impact on protein levels (Fig. 5).

In addition to acting through lipid and protein homeostasis, Tilorone may improve the functional fitness of mitochondria in the context of α-syn seeding and propagation. The observed increase in ACADS and other mitochondria-associated proteins by α-syn fibril treatment may reflect an adaptive mechanism to boost fatty acid oxidation and ATP production, which could support essential cellular functions. However, prolonged fatty acid oxidation may elevate reactive oxygen species (ROS) production, leading to oxidative stress and mitochondrial and cellular damages. It remains to be elucidated whether the molecular signature changes identified in the 3D organoid model can be seen in PD patients, particularly at an early stage of disease development [56–59].

Compared to mouse models bearing exogenously administered α-syn fibrils or α-syn transgene, the 3D organoid model as a drug screening platform offers several advantages: (1) Because organoids are derived from human iPSCs, they closely mimic human brain physiology and pathology, offering more relevant insights compared to animal models. (2) Organoids can be produced in large quantities, allowing for high-throughput screening of multiple drug candidates simultaneously, accelerating drug discovery. (3) The development and maintenance of 3D organoids are less expensive and labor-intensive compared to animal models, making it an economical option for preliminary drug testing and evaluation. (4) Using human-derived organoids also reduces ethical concerns associated with animal testing, aligning with the 3R principle (Replacement, Reduction, and Refinement) in scientific research. This system is particularly well-suited for testing candidates from drug repurposing screens, as these agents are typically well-tolerated by humans and possess favorable pharmacodynamics (PD) and pharmacokinetics (PK) properties.

However, the 3D organoid system has limitations, particularly in evaluating long-term toxicity and systemic effects. Organoids do not fully replicate the complexity of an entire living organism. Consequently, they may only capture some of the potential side effects and interactions that a drug might have in a whole-body context. Therefore, to ensure a comprehensive evaluation before advancing to clinical trials, it is essential to test Tilorone’s toxicity and efficacy in animal models after promising results in the organoid model. Animal models provide several essential insights: They help assess long-term exposure to Tilorone, identifying potential chronic toxicity and side effects that may not be apparent in organoid studies at curent time; They can reveal how Tilorone interacts with various organ systems, providing a holistic understanding of its safety profile; They offer information on how Tilorone is metabolized and distributed throughout the body, which is crucial for optimizing dosing regimens; They allow for the evaluation of behavioral and functional outcomes, which are critical for understanding the therapeutic potential of Tilorone in a living organism. Additionally, it is essential to consider other potential benefits of testing Tilorone’s action in animal models. For example, Tilorone was shown to inhibit acetylcholinesterase in vitro [41]. Since acetylcholinesterase is an enzyme responsible for breaking down acetylcholine, its inhibition may reduce the depletion of this vital neurotransmitter. If present in animals, this mechanism could help slow cognitive decline, which is a significant aspect of PD progression. Future studies delineating Tilorone’s mechanisms of action and effectiveness in vivo and its safety profile over prolonged treatment are required to fully establish its therapeutic potential for PD and related neurodegenerative disorders.

### Electronic supplementary material

Below is the link to the electronic supplementary material.

Supplementary Material 1

Supplementary Material 2

Supplementary Material 3

## Data Availability

All data generated or analyzed during this study are included in this published article and its supplementary information files.
