# Supplementary material for: Tilorone mitigates the propagation of α-synucleinopathy in a midbrain-like organoid model
Source: J Transl Med. 2024 Sep 2;22:816. doi: 10.1186/s12967-024-05551-7 (PMC11370279; doi:10.1186/s12967-024-05551-7)
Supplement: Supplementary file 3 — Supplementary Material 3 [file 12967_2024_5551_MOESM3_ESM.docx]

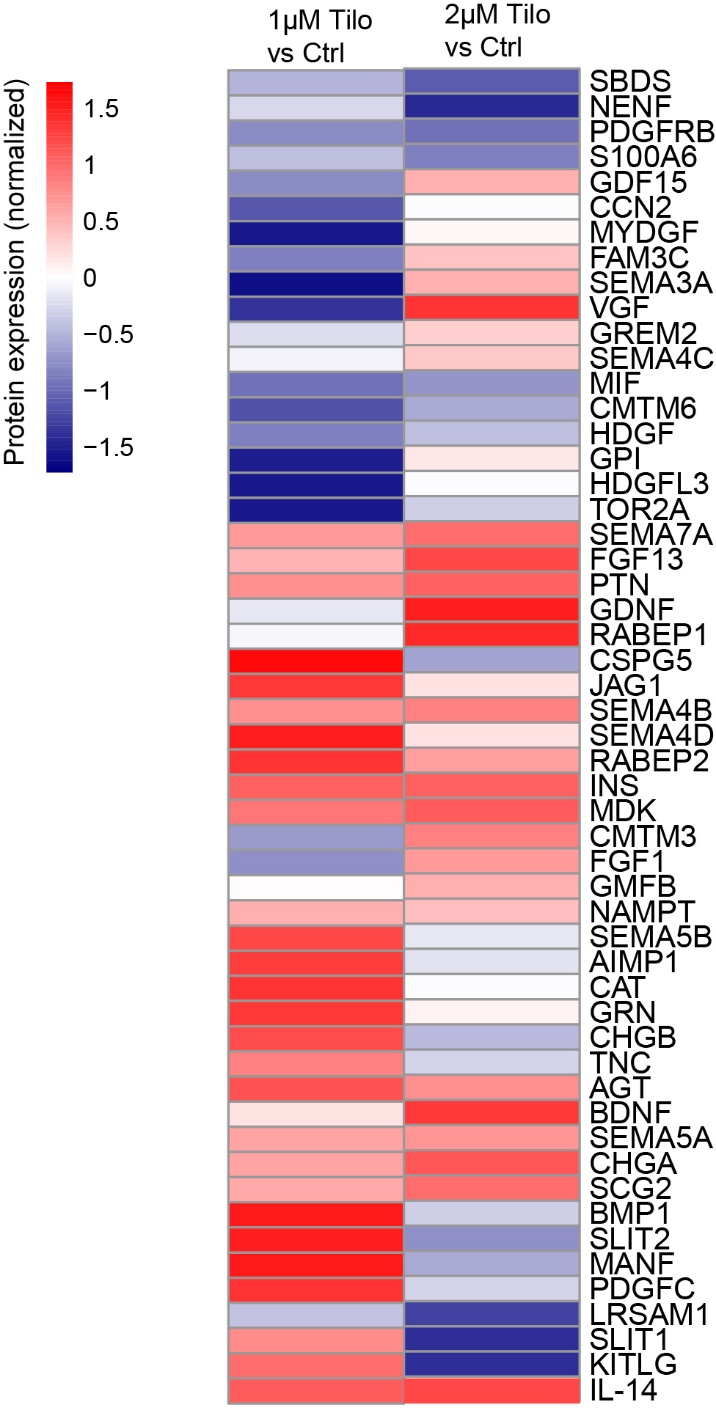


**Figure S1.** The effect of Tilorone on the levels of secretory proteins in midbrain organoids.

Heatmaps showing secretory proteins whose abundance was altered by Tilorone (Tilo) treatment at 1 µM and 2 µM. Proteins with p-value <0.05 and Log2 FoldChange >0.1 were considered as differentially regulated.
